# Ectopic expression of R3 MYB transcription factor gene *OsTCL1* in Arabidopsis, but not rice, affects trichome and root hair formation

**DOI:** 10.1038/srep19254

**Published:** 2016-01-13

**Authors:** Kaijie Zheng, Hainan Tian, Qingnan Hu, Hongyan Guo, Li Yang, Ling Cai, Xutong Wang, Bao Liu, Shucai Wang

**Affiliations:** 1 Key Laboratory of Molecular Epigenetics of MOE & Institute of Genetics and Cytology, Northeast Normal University, Changchun, 130024 China

## Abstract

In Arabidopsis, a MYB-bHLH-WD40 (MBW) transcriptional activator complex activates the homeodomain protein gene *GLABRA2* (*GL2*), leading to the promotion of trichome formation and inhibition of root hair formation. The same MBW complex also activates single-repeat R3 MYB genes. R3 MYBs in turn, play a negative feedback role by competing with R2R3 MYB proteins for binding bHLH proteins, thus blocking the formation of the MBW complex. By BLASTing the rice (*Oryza sativa*) protein database using the entire amino acid sequence of Arabidopsis R3 MYB transcription factor TRICHOMELESS1 (TCL1), we found that there are two genes in rice genome encoding R3 MYB transcription factors, namely *Oryza sativa TRICHOMELESS1* (*OsTCL1*) and *OsTCL2*. Expressing *OsTCL1* in Arabidopsis inhibited trichome formation and promoted root hair formation, and OsTCL1 interacted with GL3 when tested in Arabidopsis protoplasts. Consistent with these observations, expression levels of *GL2*, R2R3 MYB transcription factor gene *GLABRA1* (*GL1*) and several R3 MYB genes were greatly reduced, indicating that OsTCL1 is functional R3 MYB. However, trichome and root hair formation in transgenic rice plants overexpressing *OsTCL1* remained largely unchanged, and elevated expression of *OsGL2* was observed in the transgenic rice plants, indicating that rice may use different mechanisms to regulate trichome formation.

Trichome and root hair formation in the dicot plant Arabidopsis is controlled by the interplay of several transcription factors including the WD40-repeat protein TTG1 (TRANSPARENT TESTA GLABRA1)^1^, the R2R3 MYB transcription factor GL1 (GLABRA1)^2^ or WER (WEREWOLF)^3^, the bHLH transcription factor GL3 (GLABRA3) or EGL3 (ENHANCER OF GLABRA3)^4^,^5^, the homeodomain protein GL2 (GLABRA2)^6^, and seven R3 MYB transcription factors including TCL1 (TRICHOMELESS1)^7^, TCL2 (also known as CPL4 (CAPRICE-LIKE MYB4))^8^,^9^, TRY (TRYPTICHON)^10^, CPC (CAPRICE)^11^, ETC1 (ENHANCER OF TRY AND CPC1)^12^,^13^, ETC2^14^, and ETC3(also known as CPL3)^15^,^16^,^17^.

GL1 or WER, GL3 or EGL3 and TTG1 form a MBW (MYB-bHLH-WD40) transcriptional activator complex to induce the expression of *GL2*, leading to the promotion of trichome formation and the inhibition of root hair formation^6^,^18^,^19^. The same MBW complex can also induce the expression of some R3 MYB genes in plant cells^17^. R3 MYBs play a negative feedback role in the regulation of trichome and root hair formation by moving from a trichome or none root hair precursor cell to its neighboring cells, and competing with GL1 or WER for binding GL3 or EGL3, thus inhibiting the formation of the MBW complex^18^,^19^,^20^,^21^,^22^,^23^,^24^,^25^. However, not all the R3 MYB genes in Arabidopsis are activated by the MBW complex^17^, and SPL9 (SQUAMOSA PROMOTER BINDING PROTEIN LIKE9) has been shown to be able to activate *TCL1*, *TCL2* and *TRY*^8^,^26^. In addition, some of the R3 MYBs including TCL1 and TCL2 can also directly suppress the expression of *GL1*^7^,^8^. These results suggest that R3 MYBs may use different mechanisms to regulate trichome and root hair formation in Arabidopsis.

Available evidence suggests that trichome and root hair formation in other dicot plants may be controlled by similar mechanisms. For example, MYB like genes regulate trichome formation in *Mimulus guttatus* and peach^27^,^28^, expression of *GL3* in *Brassica napus* resulted in ectopic trichome formation^29^, and functional homologues of GL1 and GL2 in cotton regulate trichome formation in Arabidopsis and seed fiber development in cotton^30^,^31^,^32^,^33^. On the other hand, expression of tomato and poplar R3 MYB genes in Arabidopsis inhibited trichome formation^34^,^35^.

Single mutants of Arabidopsis R3 MYB genes have different phenotypes, however, over-expression of any of the R3 MYB genes from dicot plants inhibited trichome formation, and in some case, promoted root hair formation in Arabidopsis^7^,^10^,^11^,^21^,^34^,^35^,^36^. It remains unknown if R3 MYBs from monocot plants may also be involved in the regulation of trichome formation.

Here we report the identification and characterization of the rice R3 MYB transcription factor gene *OsTCL1* (*Oryza sativa TRICHOMELESS1*). We found that *OsTCL1* inhibited trichome formation and promoted root hair formation when expressed in Arabidopsis, however, trichome and root hair formation were largely unaffected in transgenic rice plant overexpressing *OsTCL1*, indicating that rice may use different mechanisms to regulate trichome and root hair formation.

## Results

### Identification of rice R3 MYB transcription factors

Over-expression of any of the Arabidopsis R3 MYB genes inhibited trichome formation, and in some case, promoted root hair formation^7^,^10^,^11^,^21^,^36^, expression of R3 MYB genes from other dicot plants in Arabidopsis had similar results^34^,^35^. Monocot plants are believed to have evolved from ancient dicots^37^. To investigate if R3 MYB in monocot plants may also regulate trichome formation, we decided to analyze the functions of R3 MYB transcription factors in rice, a model monocot plant.

**Figure 1:**
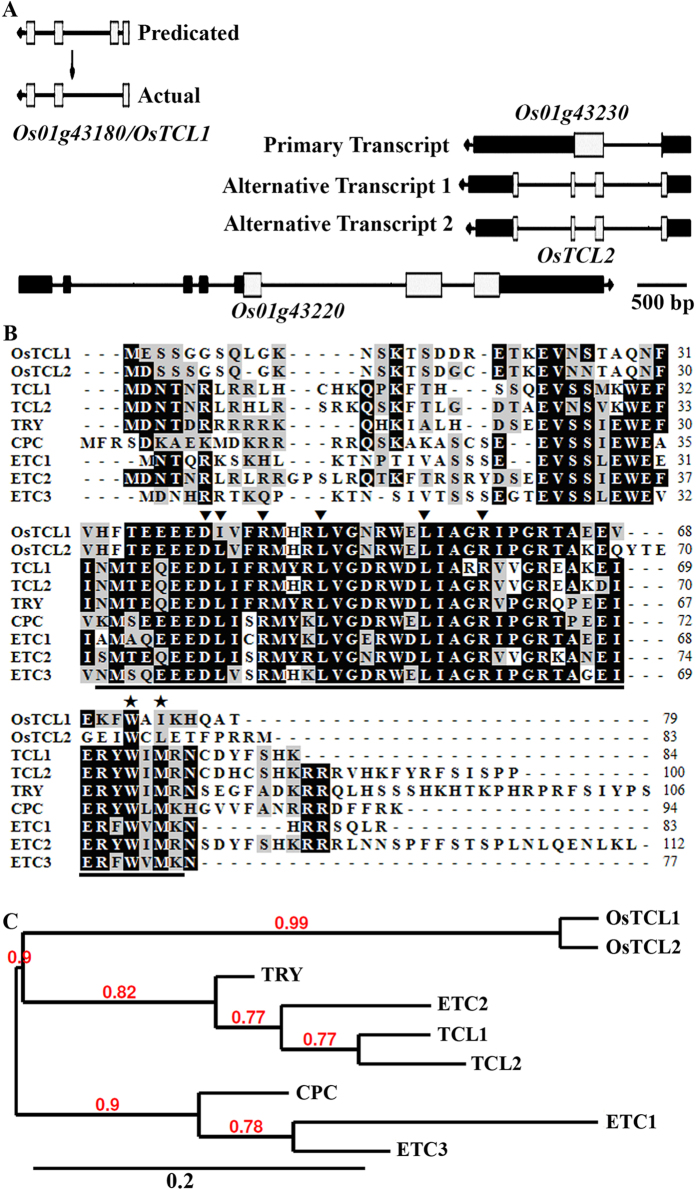
Identification of R3 MYB transcription factors in rice. (**A**) Gene structures of putative R3 MYB encoding genes in rice. OsTCL1 was encoded by *Os01g43180*, a gene predicated to have four exons, but sequencing of the amplified coding sequence indicated that it has only three exons, a gene structure similar to R3 MYB genes in Arabidopsis and poplar (http://phytozome.jgi.doe.gov/pz/portal.html). *Os01g43230*, another putative R3 MYB encoding gene, is overlapped with *Os01g43220*. It was predicated that *Os01g43220* has three transcripts, the primary transcript encodes an expressed protein with no homolog could be identified in Arabidopsis (http://phytozome.jgi.doe.gov/pz/portal.html). Two alternative transcripts of *Os01g43220* encode R3 MYB protein OsTCL2. However, we failed to amplify its coding sequence. Arrows indicate transcription direction, white boxes indicate exons, and black boxes indicate UTR regions. (**B**) Sequence alignment of rice R3 MYBs with Arabidopsis R3 MYB proteins. Identical amino acids are shaded in black, and similar amino acids are shaded in grey. The R3 MYB domain is indicated by underline. The amino acid signature [D/E]L × 2[R/K] × 3L × 6L × 3R that is required for interaction of MYB proteins with R/B-like BHLH transcription factors is indicated by arrowheads. The amino acids within the MYB domain that have been shown to be crucial for cell-to-cell movement of CPC are indicated by asterisks. (**C**) Phylogenetic analysis of rice and Arabidopsis R3 MYB transcription factors. The entire amino acid sequences of the R3 MYBs were used to generate the phylogenetic tree on Phylogeny (www.phylogeny.fr) by using “One Click” mode with default settings. Branch support values are indicated above branches. Bar indicates branch length.

By using the entire amino acid sequence of TCL1 to BLAST search the rice proteome (http://phytozome.jgi.doe.gov/pz/portal.html), and using the entire amino acid sequences of the identified rice R3 MYB transcription factors to BLAST search the rice proteome again, only the two previous reported loci, *Os01g43180* and *Os01g43230*^16^,^34^, were identified to encode R3 MYB transcription factors, and were named *OsTCL1* (*Oryza sativa TRICHOMELESS1*) and *OsTCL2*, respectively (Fig. 1A).

According to the information on phytozome, the Locus *Os01g43230* is overlapped with the Locus *Os01g43220*, and it has three different transcripts, out of them, the two alternative transcripts were predicted to encode OsTCL2 (Fig. 1A). However, we failed to amplify *OsTCL2* encoding sequences by RT-PCR.

*OsTCL1* was predicated to have four exons according to phytozome (Fig. 1A). However, after amplifying and sequencing the coding sequence of *OsTCL1*, we found it actuarially has three exons (Fig. 1A), a gene structure similar to all of the seven R3 MYB genes in Arabidopsis (http://phytozome.jgi.doe.gov/pz/portal.html).

Similar to the Arabidopsis R3 MYB proteins, nearly the entire protein of OsTCL1 is made up of a single R3 MYB domain (Fig. 1B). However, L, the second conserved amino acid in [D/E]L × 2[R/K] × 3L × 6L × 3R, the amino acid signature required for the interaction of MYB proteins with R/B-like bHLH transcription factors^38^, was replaced by amino acid I in OsTCL1 (Fig. 1B). Similarly, the amino acid M in W × M, the motif that has been shown to be required for the cell-to-cell movement of CPC^39^, was also replaced by amino acid I (Fig. 1B).

Phylogenetic analysis using full-length protein sequences of the rice and Arabidopsis R3 MYBs showed that the Arabidopsis R3 MYB formed two subgroups (Fig. 1C), as described previously^8^. OsTCL1 is paired with OsTCL2 and is closely related to one of the Arabidopsis R3 MYBs subgroup containing TRY, ETC2, TCL1 and TCL2 (Fig. 1C).

### *OsTCL1* inhibited trichome formation and promoted root hair formation when expressed in Arabidopsis

**Figure 2:**
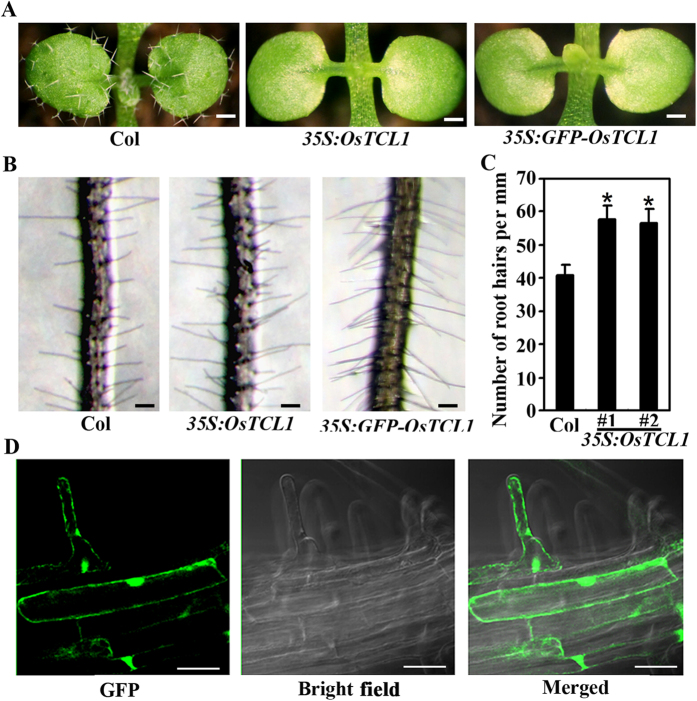
Effects of *OsTCL1* on trichome and root hair formation in Arabidopsis, and subcellular localization of OsTCL1. (**A**) Glabrous phenotypes of Arabidopsis transgenic plants expressing *OsTCL1* or *GFP-OsTCL1* under the control of the double *35S* promoter. The transgenic lines are in Col background. Pictures were taken from two-week-old soil-grown plants. Bar, 0.5 mm. (**B**) Root hair formation in Arabidopsis transgenic plants expressing *OsTCL1* or *GFP-OsTCL1* under the control of the double *35S* promoter. Pictures were taken from 10-day-old vertically grown seedlings. Bar, 0.2 mm. (**C**) Root hair density on the roots of 10-day-old Arabidopsis transgenic plant seedlings expressing *OsTCL1* under the control of the double *35S* promoter. Root hair formation on Col wild type and two independent lines were examined. Data represent the mean ± SD of 29 seedlings. *: Significantly different from Col wild type plants (P < 0.0001). (**D**) GFP fluorescence in the epidermal cells of the roots in 10-day-old *35S:GFP-OsTCL1* transgenic plant seedlings. Left panel: GFP channel, middle panel: bright field image, right panel: merged image. Bar, 10μm.

Arabidopsis R3 MYB genes inhibited trichome formation, and in some case, promoted root hair formation when overexpressed in Arabidopsis^7^,^10^,^11^,^21^,^36^, expression of R3 MYB genes from other dicot plants also inhibited trichome formation in Arabidopsis^34^,^35^. To analyze if *OsTCL1* regulates trichome and/or root hair formation when expressed in Arabidopsis, we generated transgenic Arabidopsis plants expressing *HA*-tagged *OsTCL1* under the control of the *35S* promoter (*35S:OsTCL1*). We found that expression of *OsTCL1* in Arabidopsis resulted in glabrous phenotypes in aerial parts of the plants (Fig. 2A), and increased root hairs in root (Fig. 2B,C).

Having shown that *OsTCL1* inhibited trichome formation and promoted root hair formation when expressed in Arabidopsis (Fig. 2), we wanted to further examine if OsTCL1 is the functional equivalent of the Arabidopsis R3 MYBs by testing if *OsTCL1* could rescue the mutant phenotypes when expressed under the control of the native promoter of corresponding Arabidopsis R3 MYB genes. Among the single mutants of the Arabidopsis R3 MYB genes, only three of them have trichome and/or root hair phenotypes. The *tcl1* mutants have ectopic trichome formation on the inflorescence stems and pedicels^7^, the *try* mutants have trichome clusters^10^, whereas the *cpc* mutants have increased numbers of trichome on leaves, and reduced root hairs in root^11^. So we decided to examine if OsTCL1 is the functional equivalent of TCL, TRY or CPC.

**Figure 3:**
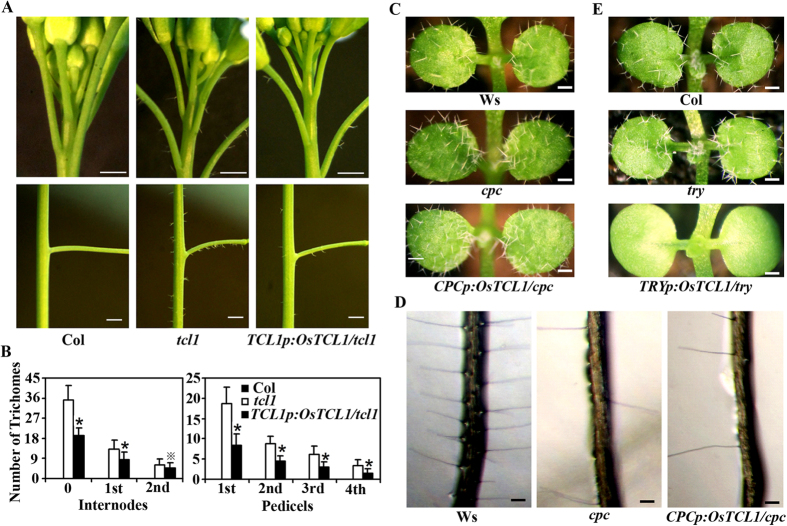
Phenotypes of transgenic plants expression of *OsTCL1* under the control of the *TCL1*, *CPC* and *TRY* native promoter, respectively, in the *tcl1*, *cpc* and *try* mutants. (**A**) Trichome formation in inflorescences of Col wild type, *tcl1* mutant, and *TCL1p:OsTCL1/tcl1* transgenic plants. Pictures were taken from five-week-old soil-grown plants. Bar, 1 mm. (**B**) Trichome density on the internode before (0) and after (first and second) the site of the first flower, and pedicels on the main inflorescence stem of wild type and transgenic plants. Data represent means ± SD of 22 plants. *Significantly different from *tcl1* mutants plants (*P < 0.0001, ^*^P < 0.05), (**C**) Trichome formation in seedlings of Ws wild type, *cpc* mutant, and *CPCp:OsTCL1/cpc* transgenic plants. Pictures were taken from two-week-old soil-grown plants. Bar, 0.5 mm. (**D**) Root hair formation in seedlings of Ws wild type, *cpc* mutant, and *CPCp:OsTCL1/cpc* transgenic plants. Pictures were taken from 10-day-old vertically grown seedlings. Bar, 0.2 mm. (**E**) Trichome formation in inflorescences of Col wild type, *try* mutant, and *TRYp:OsTCL1/try* transgenic plants. Pictures were taken from two-week-old soil-grown plants. Bar, 0.5 mm.

Transgenic plants were generated to express *OsTCL1* in the *tcl1* background under the control of the *TCL1* native promoter (*TCL1p:OsTCL1/tcl1*), in the *try* background under the control of the *TRY* native promoter (*TRYp:OsTCL1/try*), and in the *cpc* background under the control of the *CPC* native promoter (*CPCp:OsTCL1/cpc*). As shown in Fig. 3A,B, expression of *OsTCL1* under the control of the *TCL1* native promoter partially rescued the *tcl1* mutant phenotypes. Expression of *OsTCL1* under the control of the *CPC* native promoter in the *cpc* mutant background had little, if any, effect on the *cpc* mutant phenotypes (Fig. 3C,D). On the other hand, expression of *OsTCL1* under the control of the *TRY* native promoter in the *try* mutant background resulted in glabrous phenotypes (Fig. 3E).

### OsTCL1 is localized in nucleus, but also associated with plasma membrane

Having shown that expression of *OsTCL1* in Arabidopsis inhibited trichome formation and promoted root hair formation (Fig. 2), and OsTCL1 partially rescued the *tcl1* mutant phenotypes (Fig. 3), we wanted to further explore how OsTCL1 regulates trichome and root hair formation in Arabidopsis. We first examined if OsTCL1 is a nuclear protein by generating transgenic plants expressing *GFP-OsTCL1* under the control of the double *35S* promoter (*35S:GFP-OsTCL1*), and examining the subcellular localization of the fusion protein. We found that expression of *GFP-OsTCL1* in Arabidopsis inhibited trichome formation and promoted root hair formation (Fig. 2A,B), similar to that observed in the transgenic plants expressing *OsTCL1*(Fig. 2A,B), indicating that the GFP-OsTCL1 fusion protein is likely functional, thus the transgenic plant can be used to examine the subcellular localization of OsTCL1.

By examining the *GFP-OsTCL1* transgenic plants obtained, we found that GFP florescence was observed in the nucleus of the root epidermal cells, including root hairs (Fig. 2D), but GFP florescence was also observed at the plasma membrane and possible other parts of the cells (Fig. 2D).

### OsTCL1 interacts with GL3 in protoplasts

R3 MYBs regulate trichome and root hair formation in Arabidopsis by competing with GL1 or WER for binding GL3 or EGL3, and thus eliminating the formation of MBW transcriptional activator complex^20^,^21^,^22^,^23^,^24^,^25^. We have previously demonstrated that R3 MYBs from Arabidopsis and poplar interacted with GL3 in plant cells^8^,^17^,^35^, so we tested whether OsTCL1 would interact with GL3 in plant cells.

**Figure 4:**
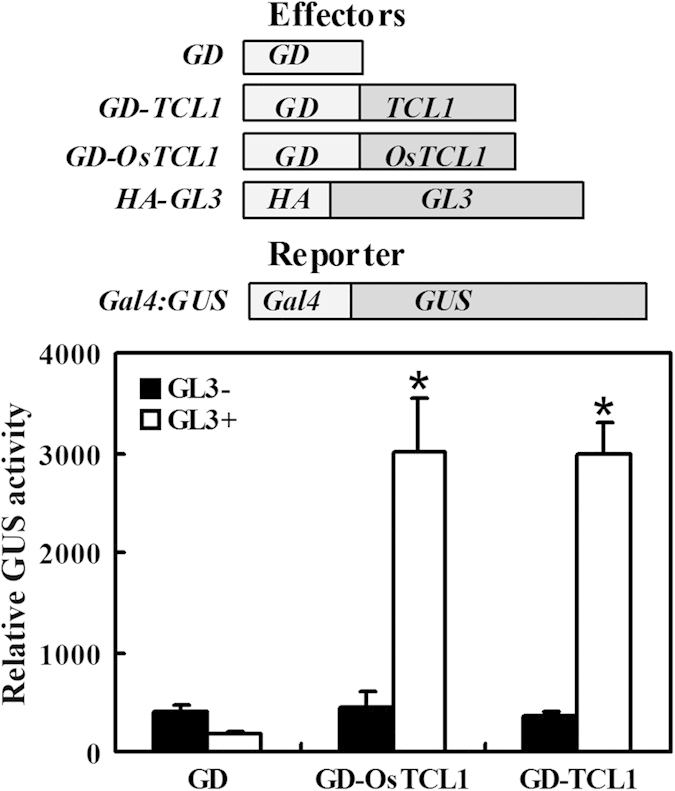
OsTCL1 interacts with GL3 in Arabidopsis protoplasts. Effector and reporter gene (diagrammed on the top of the figure) plasmids were co-transfected into protoplasts isolated from Arabidopsis rosette leaves. Transfected protoplasts were incubated in darkness for 20–22 h before GUS activity was measured. Data represent the mean ± SD of three replicates. *: Significantly different from absence of GL3 (GL3-) (P < 0.005).

Plasmids of effector gene *GL3* and GD fused OsTCL1 (*GD-OsTCL1*), together with the reporter gene *Gal4-GUS* (Fig. 4), were co-transfected into protoplasts isolated from Arabidopsis leaves. *GD* and *GD-TCL1* were used as negative and positive controls, respectively. As shown in Fig. 4, in accordance with our previously results^8^,^17^,^35^, neither GD-TCL1 nor GD activated the reporter gene in the absence of GL3. In the presence of GL3, GD-TCL1 activated the reporter gene (Fig. 4). Similarly, GD-OsTCL1 activated the reporter gene in the presence, but not the absence of GL3 (Fig. 4).

### Expression of *GL1*, *GL2* and some of the *R3 MYB* genes is down-regulated in transgenic Arabidopsis plants expressing *OsTCL1*

**Figure 5:**
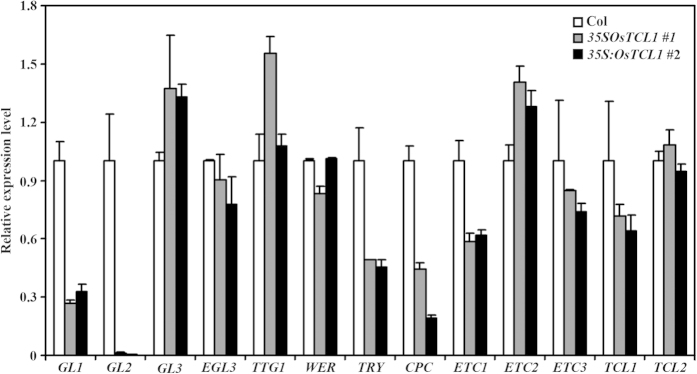
Expression of the MBW component and R3 MYB genes in Arabidopsis transgenic plants expressing *OsTCL1* under the control of the double *35S* promoter. RNA was isolated from 10-day-old seedlings of Col wild type and transgenic Arabidopsis plants, and qRT-PCR was used to examine the expression of the MBW component and R3 MYB genes. The expression of *TUBULIN2* (At5g62690) was used as a reference gene, and the expression of corresponding genes in wild type seedlings was set as 1. Data represent the mean ± SD of three replicates.

*GL2* and some R3 MYB genes are targets of the MBW transcriptional activator complex^6^,^40^. Interaction of OsTCL1 to GL3 indicates that expression of *OsTCL1* in Arabidopsis may result in the inhibition of the formation of the MBW activator complex, thus leading to the repression of *GL2* and some R3 MYB genes. To examine if this is the case, we examined the expression of *GL2* and R3 MYB genes in Arabidopsis transgenic plants expressing *OsTCL1* by using quantitative RT-PCR (qRT-PCR). As shown in Fig. 5, expression of *GL2* and R3 MYB gene *TRY*, *CPC* and *ETC1* was dramatically reduced in transgenic plants, whereas expression of the other R3 MYB genes remained largely unchanged.

We have previously shown that *GL1* is a direct target gene of TCL1^7^. To examine if OsTCL1 may also regulates the expression of *GL1*, we examined the expression of the MBW component genes in the transgenic Arabidopsis plants expressing *OsTCL1*. The results showed that the expression of *GL1* was dramatically reduced in the transgenic plants, while the expression of all other MBW component genes including *WER*, *GL3*, *EGL3* and *TTG1* remained largely unaffected (Fig. 5).

### Trichome and root hair formation in transgenic rice plants overexpressing *OsTCL1* are largely unaffected

The results described above suggest that OsTCL1 regulates trichome and root hair formation when expressed in Arabidopsis in a manner similar to the Arabidopsis R3 MYBs. Thus we further examined whether OsTCL1 may also play a role in the regulation of trichome and/or root hair formation in rice by generating transgenic rice plants expressing *OsTCL1* under the control of the double *35S* promoter.

**Figure 6:**
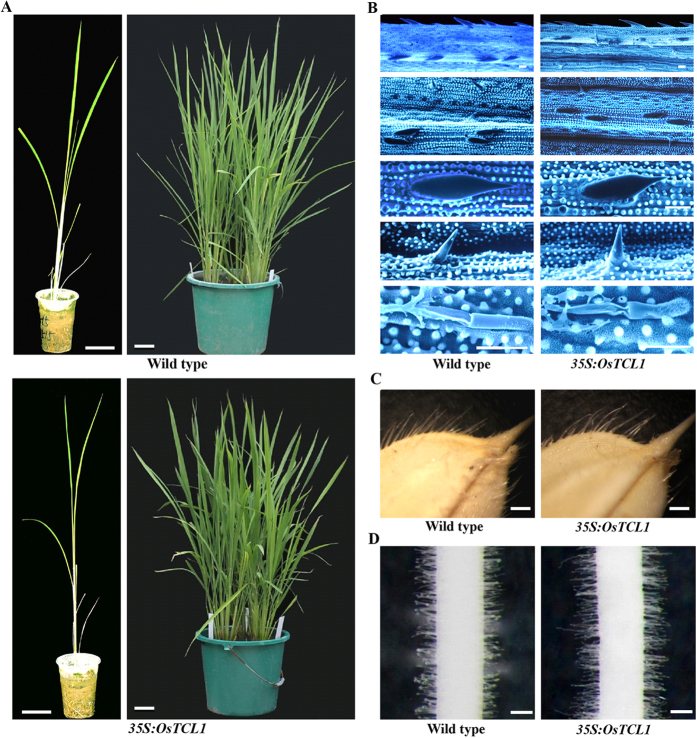
Overexpression of *OsTCL1* in rice did not affect trichome and root hair formation. (**A**) Overall morphology of wild type and transgenic rice plants overexpressing *OsTCL1* under the control of the double *35S* promoter at seedlings and adult plant stages. Pictures were taken from one-month-old and three-month-old soil-grown plants. Bar, 5 cm. (**B**) Leaf trichomes in wild type and transgenic rice plants. From up to low: leaf edge, leaf surface, and close view of macro, micro and glandular hairs. Pictures were taken under an Olympus FV1000 confocal microscope. Bar, 50 μm. (**C**) Seed trichomes in wild type and transgenic rice plants. Pictures were taken under a Motic K microscope equipped with a Canon digital camera. Bar, 0.4 mm. (**D**) Root hairs in 10-day-old seedlings of wild type and transgenic rice plants. Pictures were taken under a Motic K microscope equipped with a Canon digital camera. Bar, 0.3 mm.

**Figure 7:**
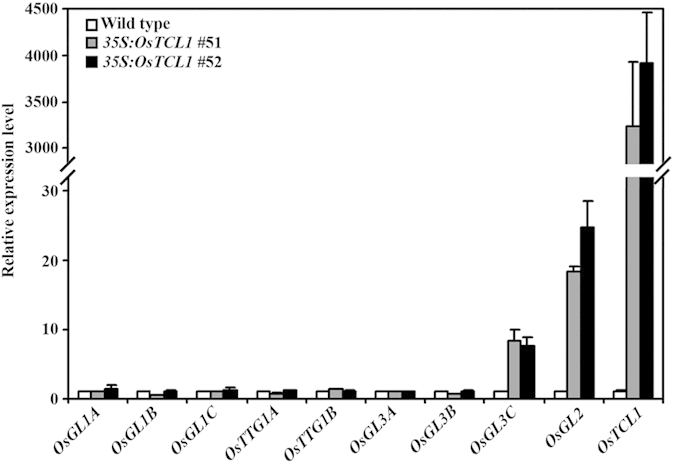
Expression of rice homolog genes of the Arabidopsis MBW component genes. Rice homologs of Arabidopsis MBW components were identified by BLAST searching the rice proteome (http://phytozome.jgi.doe.gov/pz/portal.html) using the entire amino acid sequences of Arabidopsis TTG1, GL1, GL2 and GL3. RNA was isolated from 10-day-old seedlings of wild type and transgenic rice plants, and qRT-PCR was used to examine the expression of *OsTCL1* and the rice homologs genes. The expression of *OsUBQ5* was used as a reference gene, and the expression of corresponding genes in wild type seedlings was set as 1. Data represent the mean ± SD of three replicates.

The overall morphology of the *OsTCL1* transgenic rice plants generated was largely indistinguishable from that of the wild type plants (Fig. 6A). Detailed observation under a microscope showed that trichome and root formation in the transgenic plants were also largely unaffected (Fig. 6B–D). The overexpression of *OsTCL1* in the transgenic rice plants was confirmed by qRT-PCR (Fig. 7), ruled out the possibility that unaffected trichome and root hair formation in the transgenic plants were due to low expression level of the *OsTCL1* gene. These results suggest that rice may not use the mechanisms as in Arabidopsis to regulate trichome and/or root hair formation.

To further examine this possibility, we decided to examine the expression of possible MBW component genes in the transgenic rice plants. By using the entire amino acid sequences of the Arabidopsis MBW competent transcription factors to BLAST search the rice proteome (http://phytozome.jgi.doe.gov/pz/portal.html), we identified rice genes encoding homologues of Arabidopsis transcription factor GL1, GL2, GL3 and TTG1, namely *OsGL1A* (*Os08g43550*), *OsGL1B* (*Os09g36370*), *OsGL1C* (*Os01g50110*), *OsGL2* (*Os01g55549*), *OsGL3A* (*Os04g47080*), *OsGL3B* (*Os04g47040*), *OsGL3C* (*Os07g11020*), *OsTTG1A* (*Os02g45810*) and *OsTTG1B* (*Os02g32430*). Quantitative RT-PCR analysis results showed that the expression level of *OsGL2* gene was increased in the transgenic rice plants overexpressing *OsTCL1* (Fig. 7), rather than decreased as in transgenic Arabidopsis plant expressing *OsTCL1* (Fig. 5). The expression level of *OsGL3C* gene was also increased in the transgenic rice plants overexpressing *OsTCL1* (Fig. 7). On the other hand, the expression levels of all other genes examined remained largely unchanged in the transgenic rice plants (Fig. 7).

## Discussion

In this study we report the identification and functional characterization of OsTCL1, a rice R3 MYB transcription factor. We found that when expressed in Arabidopsis, *OsTCL1* acted as a negative regulator for trichome formation and a positive regulator for root hair formation, however, trichome and root hair formation in rice overexpressing *OsTCL1* remained largely unaffected.

According to our BLAST searching results, there are only two genes loci in rice genome, *Os01g43180* and *Os01g43230*, producing R3 MYB transcription factor coding transcripts. The two genes are tandem repeat genes located on chromosome I (Fig. 1A). However, gene *Os01g43230* overlapped with gene *Os01g43220*, and it has three transcripts, with the two alternative transcripts were predicted to encode R3 MYB transcription factor. But we failed to amplify the alternative transcripts of gene *Os01g43230*.

Nevertheless, we obtained the coding sequence of gene *OsTCL1*. Though the sequencing results indicate that the gene structure of *OsTCL1* is actually similar to R3 MYB genes in dicot plant Arabidopsis and poplar (http://phytozome.jgi.doe.gov/pz/portal.html), i.e., it contains three exons, rather than four as predicted. OsTCL1 is largely consist of mainly a R3 MYB domain (Fig. 1B), similar to all the R3 MYBs in Arabidopsis and poplar^19^,^35^. Protein cellular localization results showed that OsTCL1 is nuclear protein, but it is also associated with plasma membrane (Fig. 2), possible related to cell-to-cell movement of proteins, even though the W × M motif was not full conserved in OsTCL1 (Fig. 1B). These results indicate that OsTCL1 is a R3 MYB transcription factor.

Overexpression of any of the Arabidopsis R3 MYB genes resulted in glabrous phenotypes, and in some case, promoted root hair formation^7^,^10^,^11^,^21^,^36^. Expression of R3 MYB genes from other dicot plants in Arabidopsis also inhibited trichome formation^34^,^35^, this may be explained by the fact that all R3 MYBs examined so far interacted with GL3/EGL3 in plants cells^8^,^17^,^35^, thus they have the ability to block the formation of the MBW transcriptional activator complex, which is required for the activation of *GL2*^18^,^21^,^22^,^23^,^24^,^25^.

When expressed in Arabidopsis under the controlled of the double *35S* promoter, *OsTCL1* inhibited trichome formation and promoted root hair formation (Fig. 2), suggesting that OsTCL1 is a functional R3 MYB transcription factor. Though transcript of *OsTCL2* was undetectable, based on the amino acid alignment and phylogenic analysis results (Fig. 1), it is reasonable to assume that OsTCL2, if it actually can be produced by locus *Os01g43230*, may have similar functions as those of OsTCL1. On the other hand, phenotypic complementation experiments (Fig. 3) indicate that OsTCL1 is functional similar, although it may not be equivalent to TCL1.

We showed previously that a single amino acid substitution (D/E > T/N) in [D/E]L × 2[R/K] × 3L × 6L × 3R, a conserved amino acid signature that is required for interaction of MYB proteins with R/B-like bHLH transcription factors^38^, in poplar R3 MYBs does not affect their interaction with GL3^35^. There is also a single amino acid substitution (L > I) in the conserved [D/E]L × 2[R/K] × 3L × 6L × 3R amino acid signature in OsTCL1 (Fig. 1B), however, OsTCL1 interacted with GL3 when tested in protoplasts (Fig. 4). These results suggest that OsTCL1 can block the formation of the MBW activator complex. In accordance with this, qRT-PCR results showed that the expression of *GL2*, as well as several R3 MYB genes including *TRY*, *CPC* and *ETC1* was dramatically reduced in the transgenic plants expressing *OsTCL1* (Fig. 5).

In addition to compete with GL1/WER for binding GL3/EGL3, TCL1 can also directly suppress the expression of *GL1*^7^. Quantitative RT-PCR results showed that the expression of *GL1* was also dramatically reduced in the transgenic Arabidopsis plants expressing *OsTCL1* (Fig. 5), suggesting that OsTCL1 may directly suppress the expression of *GL1*. These results suggest that OsTCL1 regulates trichome and root hair formation in Arabidopsis by a manner similar to that of the Arabidopsis R3 MYB transcription factors.

Although most of the available evidence suggests that trichome and root hair formation in dicot plants may be controlled by similar mechanisms^27^,^28^,^29^,^30^,^31^,^32^,^33^,^34^, trichome and root hair formation in tomato transgenic plants expressing *CPC* remained largely unchanged^41^, indicating that tomato and Arabidopsis may use different mechanisms to regulation trichome and root hair formation. Several different lines of evidence suggest that the monocot plant rice may also use different mechanisms to regulate trichome and root hair formation. First, overexpression of *OsTCL1* under the control of the double *35S* promoter in rice had no effects on trichome and root hair formation (Fig. 6). Second, elevated, rather than decreased expression of *OsGL2*, a rice homolog gene of Arabidopsis *GL2* was observed in transgenic rice overexpressing *OsTCL1*, and elevated expression of *OsGL3C* was also observed in transgenic rice overexpressing *OsTCL1* (Fig. 7), whereas expression of *GL3* in transgenic Arabidopsis overexpressing *TCL1* remained largely unchanged^7^. Third, several rice genes have been shown to be involved in the regulation of trichome formation in rice, including the homeobox transcription factor gene *GLR1* (*GLABROUS RICE1*, also named *OsWOX3B* (*WUSCHEL-like homeobox*), and *DEP* (*DEPILOUS*))^42^,^43^,^44^, a Histone H3K9 Methyltransferase gene *SDG714*^45^, an expressed protein coding gene *Os05g02754*^46^, and *GLR2* (*GLABROUS RICE2*), which have not yet been finally mapped, but likely encodes a zinc finger transcription factor^47^. However, none of them are homologues of the Arabidopsis transcription factors involved in the formation of the MBW transcriptional activator complex.

In summary, our results showed that OsTCL1 is a functional R3 MYB transcription factor in regulating trichome and root hair formation when expressed in Arabidopsis, but not in rice. These results suggest that rice may use different mechanisms to regulate trichome and root hair formation.

## Methods

### Identification of rice R3 MYB transcription factors

To identify rice R3 MYB transcription factor genes, the entire amino acid sequences of Arabidopsis R3 MYB transcription factor TCL1 was used to BLAST search the rice proteome (http://phytozome.jgi.doe.gov/pz/portal.html). The entire amino acid sequences of the identified rice R3 MYB transcription factors were then used to BLAST search the rice proteome until no more rice R3 MYBs were identified. Full-length amino acid sequences of Arabidopsis and rice R3 MYBs were used for phylogenetic analysis. The analysis was performed on Phylogeny (www.phylogeny.fr) using “One Click” mode with default settings. To identify rice TTG1, GL1, GL2 and GL3 transcription factor genes, the entire amino acid sequences of Arabidopsis TTG1, GL1, GL2 and GL3 transcription factors were used respectively, to BLAST the rice proteome (http://phytozome.jgi.doe.gov/pz/portal.html).

### Plant materials and growth conditions

The *Arabidopsis thaliana* (Arabidopsis) ecotype Col-0 and Ws, and Japonica rice (*Oryza sativa*) variety *Nipponbare* were used in this study. The *tcl1* and *try* mutants were in the Col-0 background^7^,^23^, and the *cpc* mutant was in the Ws background^11^.

Arabidopsis seeds were sterilized and grown on plates containing 1/2 Murashige & Skoog (MS) basal medium with vitamins (Plantmedia) and 1% (w/v) sucrose, solidified with 0.6% phytoagar (Plantmedia). Rice seeds were generated and grown in water for 10 days. Seedlings were transferred into soil pots and grown in growth rooms at 22 °C for Arabidopsis, and 28 °C for rice with a 16/8 hour photoperiod. For plant transformation, protoplasts isolation and phenotypic analysis of adult plants, Arabidopsis seeds were sown directly into soil and grown in a growth room.

### RNA isolation and quantitative RT-PCR (qRT-PCR)

Total RNA from rice was isolated as described previously for RNA isolation from poplar^48^,^49^,^50^. Total RNA from Arabidopsis seedlings was isolated using EasyPure^TM^ Plant RNA Kit (Transgene Biotech) according to the manufacturer’s instructions.

cDNA was synthesized using total RNA isolated by Oligo(dT)-primed reverse transcription using EazyScript First-Strand DNA Synthesis Super Mix (TransGen Biotech) following the manufacturer’s procedures. qRT-PCR was performed on the Applied Biosystems 7500 real time PCR System using SYBR Green/ROX Master Mix (Thermo Scientific). The primers used for qRT-PCR examination of *TCL1*, *TRY*, *CPC*, *GL1*, *GL2*, *TUBULIN2* and *OsUBQ5* have been described previously^26^,^51^,^52^. The primers for other Arabidopsis and rice genes are listed in Table 1.

### Constructs

Effect and reporter constructs used for protoplasts transfection have been described previously^7^,^8^,^17^,^40^.

To generate HA (Human influenza hemagglutinin)- or GD (Gal4 DNA binding domain)-tagged *OsTCL1*constructs for plant transformation or protoplast transfection, the full-length open-reading frame (ORF) of *OsTCL1* was amplified by RT-PCR using RNA isolated from 10-day-old rice seedlings, and cloned in-frame with an N-terminal HA or GD tag into the *pUC19* vector under the control of the double *35S* promoter of *CaMV*.

The *35S:GFP-OsTCL1* construct was cloned by replacing the GD tag in the *35S:GD-OsTCL1* construct with GFP (Green fluorescent protein). The *TCL1p:HA-OsTCL1*, *TRYp:HA-OsTCL1*, and *CPCp:HA-OsTCL1* constructs were cloned by replacing the double *35S* promoter in *35S:HA-OsTCL1* with *TCL1*, *TRY* and *CPC* promoters, respectively^7^,^11^,^13^.

Corresponding constructs in the *pUC19* vector were digested with *EcoRI* and subcloned into the binary vector *pPZP211* or *pCAMBIA1301* for Arabidopsis and rice plant transformation, respectively.

### Plant transformation and transgenic plant selection

Arabidopsis plants about five-week-old with several mature flowers on the main inflorescence were used for transformation by using the floral dip method via *Agrobacterium tumefaciens* GV3101^53^. T1 seeds were geminated on plates containing antibiotics to select transgenic plants. More than 40 transgenic lines were obtained for each of the constructs. Phenotypes of transgenic plants were examined in the T1 generation, and at least five lines with similar phenotypes were selected and confirmed in the following two to three generations. Homozygous T3 or T4 seeds from two independent lines were used for depth analysis. Expression of corresponding genes in related lines was confirmed by RT-PCR.

Transgenic rice plants overexpressing *OsTCL1* were generated by using tissue culture methods as described^54^. More than 20 transgenic lines were obtained, and confirmed T2 overexpression plants from two independent lines were used for detailed phenotypic analysis, segregated non-transgenic plants in T2 generation were used as wild type control.

### Plasmid DNA isolation, protoplast transfection and GUS activity assay

Reporter and effector plasmids were prepared using the GoldHi EndoFree Plasmid Maxi Kit (Kangwei) according to the manufacturer’s instructions. Protoplasts were isolated from rosette leaves collected from ~four-week-old Arabidopsis plants, effector and reporter plasmids were co-transfected into protoplasts, and the transfected protoplasts were incubated at room temperature for 20–22 h under darkness as described previously^17^,^40^,^55^,^56^,^57^. GUS activities were measured using a Synergy^TM^ HT microplate reader (BioTEK).

### Microscopy

Seed trichomes and root hairs in rice, trichomes and root hairs in Arabidopsis were analyzed and photographed using a Motic K microscope equipped with a Canon digital camera. Trichome formation in rice leaf, and localization of GFP-OsTCL1 proteins in transgenic Arabidopsis plants expressing *GFP-OsTCL1* were examined under an Olympus FV1000 confocal microscope.

## Additional Information

**How to cite this article**: Zheng, K. *et al.* Ectopic expression of R3 MYB transcription factor gene *OsTCL1* in Arabidopsis, but not rice, affects trichome and root hair formation. *Sci. Rep.* **6**, 19254; doi: 10.1038/srep19254 (2016).

## Figures and Tables

**Table 1:** Primers used in this study.

Primers	Sequences
*OsTCL1-Nde1F*	5′-CAACATATGGAAAGTAGCGGTGGAAG-3′
*OsTCL1-Sac1R*	5′-CAAGAGCTCTCATGTGGCTTGATGTTAATTGC-3′
*TTG1-qF*	5′-CTCTCCTTCGAGCATCCTTATC-3′
*TTG1-qR*	5′-TCCCAAAGACGGAGGAAATC-3′
*GL3-qF*	5′-GCTCATACGGCGGATAGTAAAG-3′
*GL3-qR*	5′-CAATCTCAACGACTCCTCCAAG-3′
*EGL3-qF*	5′-TGGACGACGATGTTCATTACC-3′
*EGL3-qR*	5′-TTGTGAAGCTAGACCGCTTATC-3′
*WER-qF*	5′-AGTAGTGGTGACGAAGGAAAC-3′
*WER-qR*	5′-GACCTTTGCCATGAGCTTTG-3′
*ETC1-qF*	5′-GGCTCAGGAAGAAGAGGATTTG-3′
*ETC1-qR*	5′-CCTGGAATCCTCCCAGCTATTA-3′
*ETC2-qF*	5′-ATACCAACCGTCTTCGTCTTC-3′
*ETC2-qR*	5′-AACTCCCATTCGATGCTACTC-3′
*ETC3-qF*	5′-GGATAACCATCGCAGGACTAAG-3′
*ETC3-qR*	5′-TCACAACTTCCCACTCAAGAC-3′
*TCL2-qF*	5′-GGGATACCGCAGAGGTAAATAG-3′
*TCL2-qR*	5′-ATCCCACCTATCACCAACAAG-3′
*OSTCL1-qF*	5′-AAGCCAGCTGGGAAAGAAT-3′
*OSTCL1-qR*	5′-CTTCCTCTTCTTCTGTGAAATGAAC-3′
*OsCL1A-qF*	5′-GGCAACAAGTGGTCTCTGAT-3′
*OsGL1A-qR*	5′-GATGTGCGTGTTCCAGTAGT-3′
*OsGL1B-qF*	5′-GAACGGACAACGAGATCAAGAA-3′
*OsGL1B-qR*	5′-GCCTCGAATGATATGGTGATGT-3′
*OsGL1C-qF*	5′-CTGATCAACGACGAGCAGTTAG-3′
*OsGL1C-qR*	5′-GATTCCATGACGTCTCCATGAC-3′
*OsGL2-qF*	5′-ACGACGGAGAGGGTAGTAATAA-3′
*OsGL2-qR*	5′-GCTTCCATGATCCTGATTTGTTC-3′
*OsGL3A-qF*	5′-TTGCTGATGACGAGAGTGTTC-3′
*OsGL3A-qR*	5′-GGCAAACTTGGCTTGTATCTTC-3′
*OsGL3B-qF*	5′-AGCAACTGAGGGAGCTTTAC-3′
*OsGL3B-qR*	5′-CCATTCTGTGTCTGCGAGAT-3′
*OsGL3C-qF*	5′-GAGGAAGATATGGGCCTGATTC-3′
*OsGL3C-qR*	5′-TGACTGGGTTGGATGTTGAG-3′
*OsTTG1A-qF*	5′-GGAGCATTCCACCATCTTCTAC-3′
*OsTTG1A-qR*	5′-GGCCATGTAGTGGAAGTCATAG-3′
*OsTTG1B-qF*	5′-GGCGGTCTTGATCCCATATT-3′
*OsTTG1B-qR*	5′-CCCTGAGGATCTGCAGTTTAG-3′
